# Protocol for the development of an intervention to improve the use of Point-of-caRE DiagnostICs in the management of respiraTOry tRact infectionS in primary care (the PREDICTORS study)

**DOI:** 10.12688/hrbopenres.13962.1

**Published:** 2024-11-20

**Authors:** Joseph O'Shea, Carmel Hughes, Gerard Molloy, Cathal Cadogan, Akke Vellinga, Tom Fahey, Gail Hayward, Paul Ryan, Aoife Fleming, Eimear Morrissey, Laura Cooke, Cristin Ryan

**Affiliations:** 1 School of Pharmacy and Pharmaceutical Sciences, Trinity College Dublin, Dublin, Leinster, Ireland; 2 School of Pharmacy, Queen's University Belfast, Belfast, Northern Ireland, UK; 3 School of Psychology, College of Arts, Social Sciences and Celtic Studies, University of Galway, Galway, County Galway, Ireland; 4 School of Public Health, Physiotherapy and Sports Science, University College Dublin, Dublin, Ireland; 5 Department of General Practice, Royal College of Surgeons in Ireland, Dublin, Ireland; 6 Department of Primary Care Health Sciences, University of Oxford, Oxford, England, UK; 7 Irish College of General Practitioners, Dublin, Ireland; 8 School of Pharmacy, University College Cork, Cork, Ireland; 9 Centre for Health Research Methodology, School of Nursing and Midwifery, University of Galway, Galway, Ireland; 10 Institute for Clinical Trials, College of Medicine, Nursing and Health Sciences, University of Galway, Galway, Ireland; 11 The Irish Platform for Patient Organisations, Science, and Industry (IPPOSI), Dublin, Ireland

**Keywords:** Point-of-care test, respiratory tract infection, primary care, community pharmacist, general practitioner, antibiotics, antimicrobial resistance

## Abstract

**Background:**

Antimicrobial resistance is a significant global health challenge, exacerbated by inappropriate antibiotic prescribing, particularly in primary care where up to 50% of antibiotic prescriptions prescribed by general practitioners (GPs) and dispensed by community pharmacists (CPs) are deemed inappropriate. Respiratory tract infections (RTIs) are among the most common conditions leading to GP consultations and subsequent antibiotic prescribing, much of which is inappropriate as most RTIs are viral in nature or self-limiting bacterial infections. Point-of-care tests (POCTs) have emerged as tools to improve the diagnosis and appropriate treatment of RTIs.

**Objective:**

This study aims to develop and test an intervention to improve the use of POCTs in managing RTIs involving GPs and CPs in Irish primary care, following the UK’s Medical Research Council’s (MRC) framework for complex intervention development, involving five work-packages (WPs).

**Methods:**

WP1 involves creating best practice guidance for using POCT in managing RTIs, informed by a scoping review and validated with an expert Delphi panel. This guidance will be used to define target behaviour(s) for GPs and CPs related to POCT use. WP2 explores GP and CP perceived barriers and facilitators to these behaviours using the Theoretical Domains Framework, mapping influential domains to Behaviour Change Techniques to develop draft interventions. WP3 gathers patients’ perspectives on using POCTs for RTIs. In WP4, a task group will review and finalise the intervention(s). They will consider patients’ perspectives from WP3 and assess feasibility of the intervention(s). WP5 involves a proof-of-concept study to test the feasibility of the newly developed intervention(s).

**Conclusion:**

A theoretically informed intervention(s) for using POCT(s) in the management of RTIs in primary care in Ireland will be developed and tested in a proof-of-concept study, following MRC guidance. Further refinement and larger studies will be needed to determine its effectiveness before widespread implementation.

## Introduction

Antimicrobial resistance is a widely recognised global health challenge ^1^. It results in reduced efficacy of antibacterial, antiparasitic, antiviral and antifungal drugs, making the treatment of patients difficult, costly, or even impossible ^2^. While antibiotic resistance develops naturally as bacteria adapt to antibiotic use, overuse and misuse accelerates this process ^3^. Most antibiotic prescribing (80–90%) occurs in primary care, prescribed by general practitioners (GPs) and dispensed by community pharmacists (CPs). However, up to 50% of this prescribing is deemed inappropriate ^3– 5^.

Accurately diagnosing and treating respiratory tract infections (RTIs) is particularly challenging ^6^, with large variations reported between what is prescribed and what should be prescribed, especially considering the viral and self-limiting nature of most RTIs ^7, 8^. Key drivers of antibiotic prescribing include perceived patient pressure, fear of complications of untreated infections, the perception that prescribing antibiotics is the low-risk option because of their good tolerability profile, diagnostic uncertainty, and under-estimation of antibiotic resistance by healthcare providers ^9^.

Over the last four decades, no major new types of antibiotics have made it to market ^2^. It is therefore essential to preserve the efficacy of existing drugs by minimising the development and spread of resistance to them, while efforts to develop new treatment options proceed ^2^. Antimicrobial stewardship (AMS) programmes have been established to improve the appropriate use of antibiotics and conserve the diminishing antibiotic reserve. GPs and CPs are the most important antimicrobial stewards in primary care ^10^. They are the first point of contact of patients with infections and CPs are well positioned to provide comprehensive patient care through liaison with GPs ^11^.

A systematic review and meta-analyses of 18 AMS trials found that implementation of AMS programmes by a GP-CP inter-professional team resulted in reductions in antibiotic prescribing (OR 0.93, 95%CI, 0.90-0.95) and improvements in guideline-adherent antibiotic prescribing (OR 1.72, 95%CI, 1.04-2.84) ^12^. Whilst inter-professional collaboration in primary care has a positive impact on patient care, a recent overview of reviews by Rawlinson *et al.* recommended that key barriers and facilitators at organisational and inter-individual levels should be explored, and specific contexts considered, to enhance successful implementation of inter-professional collaboration in primary care ^13^.

Some AMS interventions that have demonstrated effectiveness in reducing antibiotic prescribing include the use of delayed prescriptions ( *i.e.*, prescribed an antibiotic but advised the patient not to start taking the course unless their condition deteriorated or failed to improve after a set period) ^14^, education interventions, audit of prescribing practices and linked feedback, enhanced communication skills training for healthcare professionals, and the use of point-of-care tests (POCTs; *i.e.,* testing of patients at the time and place of care) ^15– 19^. Several POCTs can be used to improve the diagnostic certainty of RTIs ( *i.e.,* support differentiation between viral and bacterial infections in patients presenting with symptoms of RTIs) ^3^.

Rapid antigen detection tests (RADT) can be used to detect Group A *Streptococcus*, and report good sensitivity (86%; 95% CI 83–88) and specificity (96%; CI 94–97) ^20, 21^. Procalcitonin, a biomarker whose serum concentrations are elevated in response to systematic inflammation caused by bacterial infection, can be tested for in the diagnosis of sepsis ^22, 23^. A Cochrane review by Tonkin-Crine *et al.* found that procalcitonin-guided management significantly reduced antibiotic prescribing for acute RTIs in primary care ^6^. The same review reported that C-reactive protein (CRP) POCTs also significantly reduced antibiotic prescribing in patients with RTIs in primary care ^6^.

CRP POCTs have been endorsed by a European expert panel to address antibiotic overuse for lower RTIs. They suggest developing guidelines, reimbursement structures, and educational and communication tools to promote local access and uptake of CRP POCTs ^9^. While the integration of POCTs into national guidelines and clinical practice varies, countries where POCTs are widely used, including Denmark, Norway, Sweden and the Netherlands, have lower antibiotic use compared to those where POCTs are not widely implemented ^8^. However, this association is not necessarily causal. Numerous interacting factors, including pre-existing antibiotic prescribing levels, national prescribing guidelines, AMS programmes, and culturally determined healthcare provider and patient-related factors, also play a significant role ^3^.

Further research is needed to determine how POCTs should be implemented in Irish primary care for managing RTIs. Introducing new practices and services necessitates changes in both individual and collective behaviour, which requires understanding the factors that influence behaviour within the specific context ^24^. Behaviour theories, which explicitly describe the structural and psychological processes that regulate behaviour and behaviour change, are important in understanding behaviours and guiding the development of interventions that target behaviours ^24^. We propose to develop a theory-informed behaviour change POCT(s) intervention(s) for managing RTIs in primary care in Ireland, involving both GPs and CPs.

## Aim and objectives

The aim of this research programme is to develop an intervention(s) involving GPs and CPs in primary care to improve the use of POCT(s) in the management of RTIs and to test its proof-of-concept, addressing the following objectives:

- To develop best practice guidance for using POCTs in the management of RTIs in primary care, informed by a scoping review and validated using a Delphi consensus technique
- To explore GPs’ and CPs’ perceived barriers and facilitators to using POCTs in the management of RTIs in the Irish primary care context and develop a draft intervention(s)
- To explore patients’ views on the use of POCTs to guide treatment decisions in the management of RTIs in the Irish primary care context
- To obtain the views of an expert task group on the draft intervention(s), further refine the draft intervention(s) and select the final intervention(s) for proof-of-concept testing
- To test the newly developed intervention(s) in a proof-of-concept study.

## Methods

**Figure 1.**
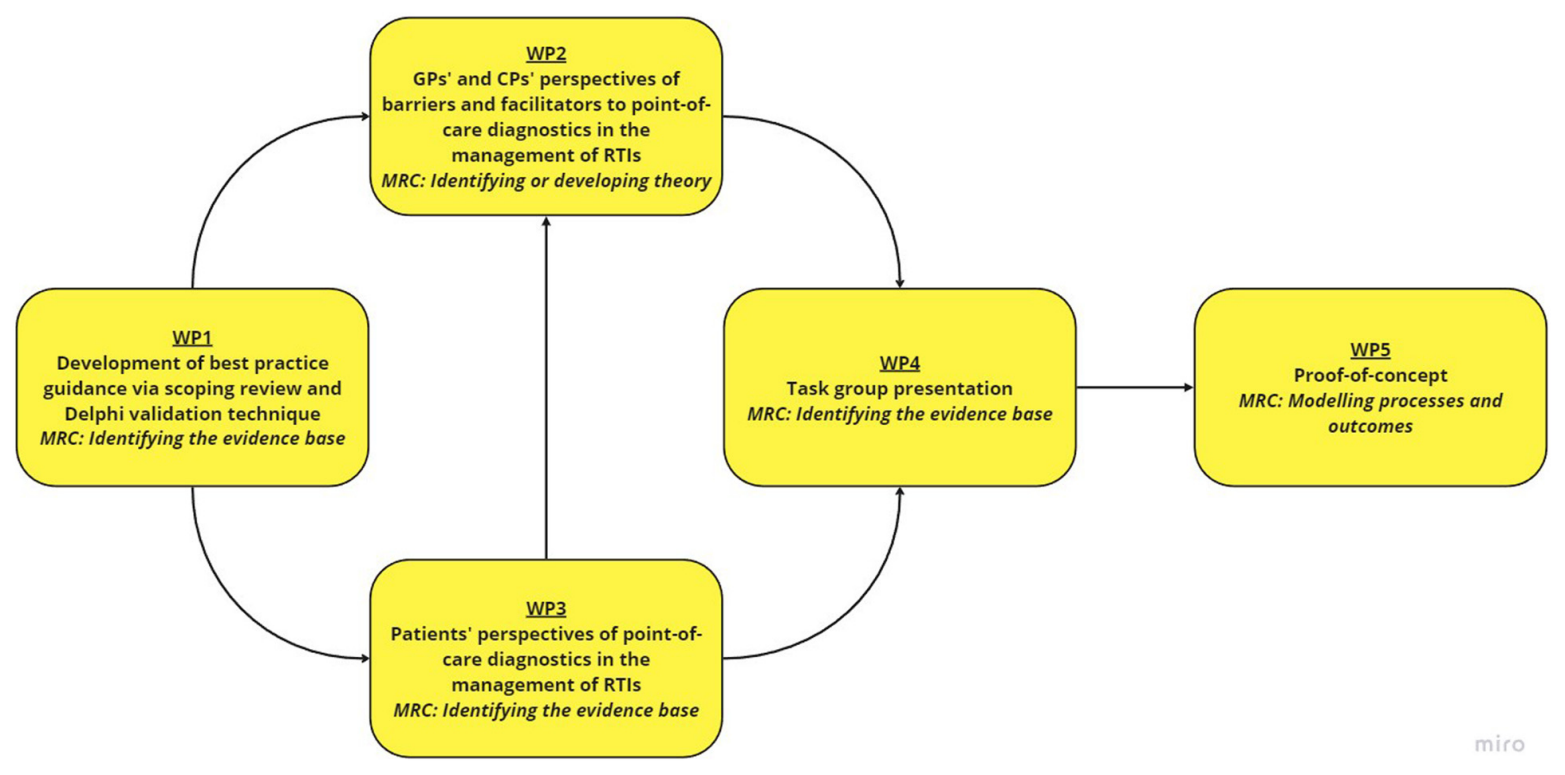
Research overview for the PREDICTORS study. Abbreviations: *CP* community pharmacist, *GP* general practitioner, *MRC* Medical Research Council, *RTI* respiratory tract infection, *WP* work-package.

This study involves five main work-packages (WPs): (i) Development of guidance; (ii) qualitative interviews with GPs and CPs; (iii) qualitative interviews with patients; (iv) intervention development with a task group, and; (v) a proof-of-concept study ( Figure 1). A Patient and Public Involvement (PPI) panel consisting of three members will be involved throughout the research programme (in particular WP3 and WP4).

### WP1: The development of best practice guidance for using POCTs in the management of RTIs in primary care

This WP involves two components, a scoping review and a Delphi consensus study.

***Scoping review***

A scoping review will be undertaken to identify published national and international evidence-based guidelines, systematic reviews, quality indicators, and individual studies that focus on the use of POCTs in the management of RTIs. The scoping review will summarise the evidence to inform the development of best practice guidance for using POCTs in the management of RTIs in primary care ( *i.e.,* by GPs and CPs) ^25^. Databases including PubMed, EMBASE, Web of Science, and CINAHL will be searched using terms such as ‘point-of-care test’, ‘antibiotics’, and ‘primary care’. The search strategy has already been developed in collaboration with the subject librarian in Trinity College Dublin. All titles and abstracts will be imported into the online platform Covidence ^®^ to manage the review process. Two reviewers will independently screen search results for eligibility and extract data using a form developed and piloted by both reviewers. Any disagreements during screening and data extraction will be resolved through discussion with a third reviewer.

***Delphi consensus study***

The findings of the scoping review will be used to inform draft best practice guidance. This will be developed by members of the team following guidance from the Appraisal of Guidelines for Research and Evaluation (AGREE) II Instrument ^26^. A Delphi consensus technique, a long-established method which allows informed decision-making through obtaining consensus from expert opinion, will then be used to validate this initial draft and to agree on its content ^27– 29^.

A Delphi panel, consisting of national clinical experts (n = 20) including respiratory physicians, microbiologists, GPs, and CPs with special interests in respiratory disease, POCTs, and AMS will be purposively recruited from the research team’s professional network. Each panel member will be provided with the best practice guidance statements in a web-based questionnaire format ( *i.e.,* Qualtrics ^®^^30^) and will be asked to agree on the content of the guidance by independently rating their level of agreement with each guidance statement using a 5-point Likert scale (1= strongly disagree, 5 = strongly agree).

Members of the Delphi panel will have the opportunity to provide commentary on each guidance statement to make suggestions for change. Statements with an overall lower quartile of ≥4 will be retained for inclusion in the final guidance, whilst those with an overall upper quartile of ≤2 will be removed ^31^. Statements with an interquartile range that includes 3 will be reviewed within the core research team, modified and recirculated for review by the expert panel in a second round Delphi questionnaire ^31^. In this second round, panel members will receive their own individual and group responses. Following further review, only those guidance statements with an overall lower quartile of ≥4 will be included. It is anticipated that three Delphi rounds will be required to achieve consensus on the final content of the guidance.

### WP2: Qualitative interviews with GPs and CPs to explore their views and perceived barriers and facilitators to using POCTs in the management of RTIs in primary care

This WP will explore the views of GPs and CPs regarding the use of POCT(s) in the management of RTIs. The Consolidated Criteria for Reporting Qualitative Research (COREQ) will be used throughout and in the reporting of this WP ^32^.

***Study design***

A qualitative study design, using individual semi-structured interviews, was chosen as this methodology will facilitate a detailed examination of the barriers and facilitators to the behaviour under investigation, *i.e.,* using POCT(s) in the management of RTIs by GPs and CPs. The exact specificity of the behaviour pertaining to using POCTs in the management of RTIs will be defined based on the best practice guidance produced in WP1, following guidance from Atkins *et al.* ^24^ If multiple behaviours are identified from the guidance, we will prioritise behaviours following Atkins *et al.*’s recommendations and select a maximum of two behaviours to target for each professional group (GPs and CPs).

In selecting the behaviour to target, the following will be considered: (i) How modifiable the behaviour is likely to be; (ii) how central it is in bringing about the desired change in clinical practice; (iii) probable/potential positive or negative effects on other related behaviours if change occurs; and (iv) its amenability to measurement ^24^. The behaviours will be explored using the Theoretical Domains Framework (TDF) V2.0 and then mapped to Behavioural Change Techniques (BCTs), which are the active ingredients of behaviour change interventions ( further details provided below). This approach has been used in the development of other complex interventions targeting healthcare professionals’ behaviour ^33– 36^.

***Sampling and recruitment of GPs and CPs***

GPs working in general practice and pharmacists working in the community setting, who have prescribed/dispensed antibiotics for RTIs will be eligible for inclusion. The Health Research Board (HRB) Primary Care Clinical Trials Network (CTN) will facilitate the recruitment of GPs by initiating contact between prospective GP participants and the researcher. Once GPs are recruited, they will assist in recruiting CPs. It is anticipated that two GPs and two CPs will be recruited per Community Health Organisation (CHO) in which they work. CHOs in Ireland are regional structures established by the Health Service Executive to deliver health and social care services at a local level. There are nine CHOs across the country, each responsible for providing a range of primary care, social care, mental health, and community health services to their respective populations.

With nine CHOs, we expect to recruit 18 GPs and 18 CPs in total. This approach to recruitment will ensure geographical diversity and that a mixture of GPs and CPs working in urban and rural areas are recruited. Furthermore, we will strive for gender balance in our recruitment. Data analysis will be conducted in tandem with data collection; therefore, recruitment will continue until data saturation is reached. Participants will be remunerated for their time (€100 per interview). Written, informed consent will be required, after which the researcher (O’Shea) will schedule the online interview ( *e.g.,* Microsoft Teams) at a date and time best suited to the participant and the researcher.

***Topic guides for GP and CP interviews***

Separate topic guides will be developed for GPs and CPs reflecting the specificity of the target behaviours as noted above. The guides will be largely based on the TDF V2.0, and informed by existing literature on barriers and facilitators to POCT use in primary care ^22, 37– 39^. The TDF V2.0 integrates insights from 33 behaviour change theories into 14 key domains (knowledge; skills; social/professional role and identity; beliefs about capabilities; beliefs about consequences; reinforcement; intentions; memory, attention and decision processes; environmental context and resources; social influences; emotion; optimism; goals; and behavioural regulation), each representing distinct aspects of behaviour and can be used to identify barriers and facilitators to components of behaviour ^24^.

It is possible that not all of the 14 domains will be considered relevant to the target behaviour by the research team and therefore some may be omitted from the topic guides. Open questions will be asked for each domain followed by prompts and cues to probe more deeply to explore barriers and facilitators to each domain. It is anticipated that each interview will last approximately 30–40 minutes. Pilot interviews with GPs and CPs will be undertaken to ensure clinical relevance. Members of the team experienced in behavioural change will also review the GP and CP topic guides. Participants will also be asked their views on potential POCT intervention components, and how GPs and CPs could work together to deliver these.

***Data management and analysis***

Interviews will be audio and video recorded, transcribed verbatim using a transcription service, anonymised, and entered into NVivo ^®^^40^. Codes will be assigned to differentiate between GP and CP participants, together with a two-digit identification number. All identifiers will be removed during transcription and only anonymised transcripts will be available to the research team for analysis using NVivo ^®^. Data analysis will be conducted in tandem with data collection; therefore, the topic guides will undergo iterative development and refinement. All interviews transcripts will be analysed by the researcher (O’Shea), with a 20% random sample also reviewed by the Principal Investigator (Ryan). Analysis will follow the framework method ^41^, with the TDF as the framework. Further content analysis of each domain will be undertaken to explore barriers and facilitators within each domain.

***Development of draft interventions***

The TDF domains identified as feasible intervention targets will be mapped to BCTs using the BCT Ontology (BCTO) ^42^. The BCTO provides a more comprehensive and structured framework for categorising and understanding BCTs (the active ingredients of behaviour change interventions), representing specific strategies designed to modify behaviour ^42^. Cane *et al.*’s mapping matrix will be used in this mapping process ^43^, ensuring that each selected TDF domain is translated into actionable BCTs within the BCTO framework ^43, 44^. Operationalising BCTs into actionable intervention components for community pharmacy and general practice will be undertaken once WP3 is completed, so that patients’ perspectives can be considered. The research team will convene to review results, resolve discrepancies, and determine how to implement the identified BCTs to develop draft intervention(s) aimed at improving the use of POCT(s) in the management of RTIs in primary care.

### WP3: Qualitative interviews with patients to explore their views on receiving POCTs to guide treatment in the management of RTIs

A qualitative study to explore patients’ views on receiving POCTs to guide treatment in the management of RTIs will be undertaken. The COREQ will also be used throughout and in the reporting of this WP ^32^.

***Sampling and recruitment of patients***

Patients/carers (aged 18 years or older) will be eligible to take part if they have previously received an antibiotic for a RTI or if someone they care for (no age limits) has been given an antibiotic for a RTI. GPs and CPs participating in WP2 will be asked to provide study information to potential patient/carer participants, and instruct them to contact the researcher (O'Shea) if they are interested in taking part. It is intended that this approach to recruitment will ensure geographical diversity and that a mixture of patients/carers from urban and rural areas are recruited. We anticipate that 15 patients/carers will provide sufficient information power for WP3 ^45^. Participants will be remunerated for their time (€100 per interview). Written, informed consent will be required, after which the researcher (O’Shea) will schedule the online interview ( *e.g.,* Microsoft Teams) at a date and time best suited to the participant and the researcher.

***Topic guide for patient interviews***

The topic guide and study documentation ( *e.g.,* participant information leaflet) will be co-developed by the researcher (O’Shea), Principal Investigator (Ryan), and PPI panel with input from members of the wider team. This guide will be informed by existing literature on barriers and facilitators to POCT in primary care ^46– 48^. It is anticipated that each interview will last approximately 30–40 minutes. Data analysis will be conducted in tandem with data collection; therefore, the topic guides will undergo iterative development and refinement. Open questions will be asked followed by prompts and cues to probe more deeply to explore participants’ views on and experience with POCTs for managing RTIs. Topics covered will include opinions on primary care, attitudes toward using POCTs for RTIs, patient expectations, and how results should be communicated.

***Data management and analysis***

As per WP2, interviews will be audio and video recorded, transcribed verbatim using a transcription service, anonymised, and entered into NVivo ^®^. All transcripts will be analysed by the researcher (O’Shea), with a 20% random sample also reviewed by the Principal Investigator (Ryan) and PPI collaborator (Cooke). Patient transcripts will undergo thematic analysis, involving familiarisation, initial coding, grouping of codes into emerging themes, and assigning overarching themes. The analysis team will meet to discuss results, resolve any disagreements in interpretation, and review the findings with our PPI panel, ensuring all considerations are addressed when finalising the draft intervention(s) (see WP2 Development of the draft interventions for further details).

### WP4: Development of POCT intervention(s) with an expert task force to improve the management of RTIs in primary care

Draft interventions (developed following data analysis in WP2 and WP3) will be presented to task groups (see below) to obtain their views on the interventions, and to aid selection of the final intervention(s) for proof-of-concept testing. Task groups are a hybrid focus group intended to produce a set of principles for action grounded in task group members’ experiences and generate qualitative data ^49, 50^. This approach will ensure that the interventions are grounded in real world experiences of the healthcare professionals involved and ensure a patient-centred approach to the interventions adopted. The task group will be co-chaired by the researcher (O’Shea) and a PPI collaborator (Cooke). Non-jargonistic language will be used throughout, and all participants will receive a €100 honorarium for participation.

***Task group formulation and function***

A task group involving GPs, CPs, our PPI panel, and members of the team will be established. GPs (n = 2) and CPs (n = 2) will be recruited from those who participated in WP2. Patient and carer representatives will include our PPI panel (n = 3). Draft interventions derived from the findings of WP1 and WP2 will be identified and prioritised through discussions within the research team. These potential interventions will be presented to task group members (including our PPI panel) at a meeting dedicated to assessing their feasibility in terms of content and delivery. This evaluation will employ the APEASE criteria (Acceptability, Practicability, Effectiveness/cost-effectiveness, Affordability, Side-effects/safety, Equity), which were developed to guide context-based decisions on intervention content and delivery ( Table 1) ^51^. Group discussions will be used to resolve discrepancies and reach agreement.

**Table 1.** The APEASE criteria for assessing interventions, intervention components and ideas with example questions (adapted from 51). This table outlines the APEASE criteria (Affordability, Practicability, Effectiveness, Acceptability, Side-effects/safety, Equity), including example questions for applying these criteria during the assessment of interventions and their components. Adapted from reference 51.

APEASE criteria	Example of potential questions
**A**cceptability	▪ How far is it acceptable to key stakeholders?
**P**racticability	▪ Can it be implemented at scale within the intended context, material and human resources? ▪ What would need to be done to ensure that the resources and personnel were in place, and is the intervention sustainable?
**E**ffectiveness	▪ How effective is the intervention in achieving the policy objective(s)? ▪ How far will it reach the intended target group and how large an effect will it have on those who are reached?
**A**ffordability	▪ How far can it be afforded when delivered at the scale intended? ▪ Can the necessary budget be found for it? ▪ Will it provide a good return on investment?
**S**ide effects & safety	▪ What are the chances that it will lead to unintended adverse or beneficial outcomes?
**E**quity	▪ How far will it increase or decrease differences between advantaged and disadvantaged sectors of society?

***Data management and analysis***

Detailed notes of the task group discussion will be taken by the researcher (O’Shea) and PI (Ryan). All feedback and suggestions put forth by task group members regarding the proposed intervention(s) will be carefully reviewed. Suggestions deemed unfeasible will be excluded from further consideration. A real-time summary and feedback on key discussion points will be provided to all task group members at the conclusion of the meeting. Subsequently, a comprehensive description of the newly developed intervention(s) will be drafted and circulated among the task group for final review and commentary. The research team will then meet online to reach a consensus on the final components of the intervention(s) that will advance to proof-of-concept testing (WP5).

### WP5: A proof-of-concept study to test the newly developed intervention(s)

This WP will assess the proof-of-concept of the newly developed intervention(s) from WP4, depending on the findings from WP1-WP3. A separate protocol for the proof-of-concept study will be developed in accordance with the SPIRIT (Standard Protocol Items: Recommendations for Interventional Trials) guidelines. Given the study's adaptive nature and its reliance on findings from previous WPs to shape the design, we cannot provide precise details of the study’s methodology at this stage. However, prior to patient enrolment, the proof-of-concept study will be registered in a WHO-recognised public repository to meet transparency and ethical standards. This approach ensures compliance with regulatory requirements, as recommended by the WHO International Clinical Trials Registry Platform (ICTRP) and the SPIRIT guidelines. A completed SPIRIT checklist will also be submitted to an online repository as extended data.

It is anticipated that the newly developed intervention(s) will be tested in two settings (community pharmacy and general practice), recruited from the GPs and CPs involved in WP2. The researcher (O’Shea) will recruit and visit each site and present an overview of the POCT intervention(s). Training and patient information leaflets will be provided as required. To assess the fidelity of the newly developed intervention(s), it is likely that participating GPs and CPs will be requested to audio record their consultations with consenting patients, which will then be transcribed and content analysed. Descriptive analysis will be conducted on the collected data. Additionally, it is intended that all participating GPs, CPs, and patients will be invited to participate in semi-structured interviews to provide feedback on the newly developed intervention(s), highlighting what aspects worked well, areas for improvement, and suggested changes. The obtained data will be analysed descriptively and narratively, synthesised and presented to members of the team for further discussion and refinement of the intervention(s) approach.

## Discussion

In light of antibiotic overprescribing and increasing antibiotic resistance, the development of an intervention(s) to improve the use of POCT(s) in the management of RTIs in primary care is critically important. This work describes the initial development phase of an evidence-based and theory-informed POCT intervention(s) to foster AMS in the management of RTIs considering the views and experiences of key stakeholders ( *i.e.,* GPs, CPs and patients/carers) in ways that are effective and feasible in the Irish primary care setting. This study will identify how GPs and CPs can work together to successfully implement AMS, highlight the challenges of introducing POCT(s) in primary care, and develop best practice guidance for using POCT(s) in the management of RTIs. It will also identify barriers and facilitators to implementation, gather patient perspectives on POCT(s) use, analyse the behavioural changes required to improve its use, and ultimately design and test POCT intervention(s) for managing RTIs in Irish primary care. This study could have the potential to optimise antibiotic prescribing, safeguard patients and prevent antibiotic resistance through collaborative antibiotic decision-making and care by GPs and CPs.

## Ethics and consent

Ethical approval will be obtained for all studies from the School of Pharmacy & Pharmaceutical Sciences Research Ethics Committee, Trinity College Dublin, and the Irish College of General Practitioners, Dublin, for WP5. To date, ethical approval has been received for WP1, WP2, and WP3. Since the design of WP4 and WP5 will be shaped by the outcomes of the earlier work-packages, obtaining ethical approval for these components is not yet feasible. Once the relevant information becomes available, ethics applications for WP4 and WP5 will be promptly submitted.

### Study status

WP1 – Approved by the School of Pharmacy & Pharmaceutical Sciences Research Ethics Committee, Trinity College Dublin. Currently finalising the analysis of Delphi questionnaire round 2.

WP2 – Approved by the School of Pharmacy & Pharmaceutical Sciences Research Ethics Committee, Trinity College Dublin.

WP3 – Approved by the School of Pharmacy & Pharmaceutical Sciences Research Ethics Committee, Trinity College Dublin.

WP4 – The study design will be informed by the outcomes of WP1-3. Once the necessary data is available, an ethics application will be submitted promptly.

WP5 – The study design will be informed by the outcomes of WP1-3. Once the necessary data is available, an ethics application will be submitted promptly.

### Consent statement

All participants will be required to provide written informed consent prior to participation in the studies outlined in this protocol, confirming their understanding of each study's purpose, procedures, and any potential risks and benefits associated with their involvement.

## Data Availability

No data are associated with this article.
