# Supplementary material for: Protocol for the development of an intervention to improve the use of Point-of-caRE DiagnostICs in the management of respiraTOry tRact infectionS in primary care (the PREDICTORS study)
Source: HRB Open Res. 2025 Mar 7;7:73. Originally published 2024 Nov 20. [Version 2] doi: 10.12688/hrbopenres.13962.2 (PMC11890267; doi:10.12688/hrbopenres.13962.2)
Supplement: Peer review history

# Reviewer report: Reviewer response for version 2

**DOI:** 10.21956/hrbopenres.15500.r46333

**Published:** 2025-03-11

**Authors:** Sajal Saha

**Affiliations:** 1 Deakin University, Geelong, Australia

The authors have addressed the comments.

Is the study design appropriate for the research question?

Yes

Is the rationale for, and objectives of, the study clearly described?

Yes

Are sufficient details of the methods provided to allow replication by others?

Yes

Are the datasets clearly presented in a useable and accessible format?

Not applicable

Reviewer Expertise:

antibiotic stewardship in primary care, GP-pharmacist care model, point-of-care testing

I confirm that I have read this submission and believe that I have an appropriate level of expertise to confirm that it is of an acceptable scientific standard.

# Reviewer report: Reviewer response for version 2

**DOI:** 10.21956/hrbopenres.15500.r46334

**Published:** 2025-03-08

**Authors:** Guido Schmiemann

**Affiliations:** 1 University of Bremen, Bremen, Germany

Thanks for the changes made. All my comments have been taken into account and I have no further remarks.

Is the study design appropriate for the research question?

Yes

Is the rationale for, and objectives of, the study clearly described?

Yes

Are sufficient details of the methods provided to allow replication by others?

Partly

Are the datasets clearly presented in a useable and accessible format?

Not applicable

Reviewer Expertise:

GP with experience in guideline development, health services research

I confirm that I have read this submission and believe that I have an appropriate level of expertise to confirm that it is of an acceptable scientific standard.

# Reviewer report: Reviewer response for version 1

**DOI:** 10.21956/hrbopenres.15318.r43347

**Published:** 2024-12-27

**Authors:** Sajal Saha

**Affiliations:** 1 Deakin University, Geelong, Australia

The PREDICTORS study seems novel in Irish primary care that develops and tests an intervention to improve the use of POCTs in RTIs by GPs and CPs. 

 The study protocol has considered scientifically sound and robust methodologies for the development of the intervention.  There is only one area of concern to me.

 The POCT implementation model remains unclear. Is the intervention development driven by GP-pharmacist collaborative implementation of POCT testing or POCT use separately in GP and pharmacy settings or both? How would author interpret, use and report those data in terms of the settings? 

 Whether the guideline that will be developed would be specific to settings (GP/pharmacy) or implementation model or in general?. A clear reporting may enhance clarity and understanding of the development and testing.

Is the study design appropriate for the research question?

Yes

Is the rationale for, and objectives of, the study clearly described?

Yes

Are sufficient details of the methods provided to allow replication by others?

Yes

Are the datasets clearly presented in a useable and accessible format?

Not applicable

Reviewer Expertise:

antibiotic stewardship in primary care, GP-pharmacist care model, point-of-care testing

I confirm that I have read this submission and believe that I have an appropriate level of expertise to confirm that it is of an acceptable scientific standard.

# Response

**Published:** 2025-02-28

**Authors:** Joseph O'Shea

**Affiliations:** Trinity College Dublin School of Pharmacy and Pharmaceutical Sciences, Dublin, Leinster, Ireland

Dear Dr Saha,

 Thank you for taking the time to review our submission. I have addressed your comments in the revised version that has been submitted. To summarize your comments:

- Clarify whether the intervention focuses on GP-CP collaboration, independent POCT use in each setting, or both.
- State explicitly how data will be analyzed and reported based on the setting.
- Clarify whether the developed guidelines will be setting-specific (GP/pharmacy) or generalizable across both settings.

These have been addressed. The description of the POCT implementation model now acknowledges that WP4 and WP5 will be shaped by earlier findings, allowing for a range of potential intervention models, including collaborative or independent GP-CP practice. The guideline specificity section has been updated to reflect that the Delphi study will determine where and by whom POCT should be used, with the final guidance incorporating only consensus-based statements while maintaining POCT-specific recommendations.

 Kind regards, 

 Dr Joseph O'Shea

# Reviewer report: Reviewer response for version 1

**DOI:** 10.21956/hrbopenres.15318.r43345

**Published:** 2024-12-02

**Authors:** Guido Schmiemann

**Affiliations:** 1 University of Bremen, Bremen, Germany

**Review of the PREDICTORS Study Protocol**

 Thank you for the opportunity to review the study protocol of the PREDICTORS study. The study aims to develop and test an intervention designed to enhance the antibiotic prescribing process for respiratory tract infections using point-of-care tests (POCT).

 Influencing the antibiotic prescribing process is a complex intervention; accordingly, the proposed study encompasses multiple aims. These aims address various perspectives, and the development of a new intervention is grounded in theoretical models suited for the study's objectives. Overall, the objectives, proposed aims, and methods are well articulated, resulting in a scientifically sound description. Since the study has already commenced and changes to the process may no longer be feasible, my suggestions primarily focus on enhancing the discussion and presentation of the results.

**Background:**

- I recommend providing context for the study by including information on prescribing rates in Ireland, which are slightly above the EU average ( https://www.ecdc.europa.eu/sites/default/files/documents/AER-antimicrobial-consumption.pdf).
- While some point-of-care tests are mentioned, the authors might consider elaborating on which POCTs are currently approved or used in primary care settings in Ireland.
- It appears that economic aspects are not addressed in the study or the proof-of-concept phase. Given the existing evidence regarding CRP tests in Ireland (Fawsitt CG. et al, 2022 [Ref-1]), this information could strengthen the background or discussion sections.

**Methods:**

- To develop best practice guidance for utilizing POCTs in managing RTIs in primary care, a scoping review that assesses the literature will be employed. I suggest also searching for grey literature, including projects already conducted in various healthcare systems in Ireland, as they may reveal valuable lessons not captured within the published literature.
- When recruiting participating GPs and Community Pharmacists (CPs), gender is mentioned as a potential influencing factor. It would be beneficial to consider additional factors, such as age and urban versus rural settings, as they may impact familiarity with or accessibility to POCTs.
- The study plans to assess patient perspectives through qualitative interviews. The content analysis of these interviews will be conducted by one researcher, with a random sample reviewed by a second researcher. Please clarify whether thematic analysis, coding, etc., will solely be performed by the primary researcher. The protocol mentions an analysis team; how does their role influence the final coding process? Is the final coding completed only after the analysis team has consented to the coding framework? Additional clarity on this process would be appreciated.

**Discussion:**

- In discussing the development of the intervention, it is important to consider the role of payers in the success of any future intervention. Are payer perspectives incorporated into the development of the intervention? Understanding this aspect could be crucial for addressing the needs and dynamics of the Irish healthcare system.

Is the study design appropriate for the research question?

Yes

Is the rationale for, and objectives of, the study clearly described?

Yes

Are sufficient details of the methods provided to allow replication by others?

Partly

Are the datasets clearly presented in a useable and accessible format?

Not applicable

Reviewer Expertise:

GP with experience in guideline development, health services research

I confirm that I have read this submission and believe that I have an appropriate level of expertise to confirm that it is of an acceptable scientific standard, however I have significant reservations, as outlined above.

## References

[1] : A cost-effectiveness and budget impact analysis of C-reactive protein point-of-care testing to guide antibiotic prescribing for acute respiratory tract infections in primary care settings in Ireland: a decision-analytic model. *Fam Pract* .2022;39(3) : 10.1093/fampra/cmab123 389-397 10.1093/fampra/cmab123 34591966

# Response

**Published:** 2025-02-28

**Authors:** Joseph O'Shea

**Affiliations:** Trinity College Dublin School of Pharmacy and Pharmaceutical Sciences, Dublin, Leinster, Ireland

Dear Dr Schmiemann,

 Thank you for taking the time to review our submission. I have addressed your comments in the revised version that has been submitted. To summarize your comments:

 1. Background:

- Prescribing Rates in Ireland: Add a sentence referencing the ECDC report on Ireland’s antibiotic prescribing rates being slightly above the EU average. Explain how this context supports the need for interventions like POCT.
- Approved POCTs in Irish Primary Care: Expand the section on POCTs to clarify which ones are currently approved or used in primary care in Ireland.
- Economic Considerations: Acknowledge that economic factors are not the primary focus of this study but reference existing research (e.g., Fawsitt et al., 2022) in the discussion to highlight the economic feasibility of CRP POCTs in Ireland.

2. Methods:

- Grey Literature in Scoping Review: Add a statement that grey literature, including national projects and reports, will be reviewed alongside peer-reviewed literature.
- Recruitment Factors Beyond Gender: Clarify that in addition to gender, recruitment will aim for diversity in age and urban/rural practice settings.
- Clarification on Qualitative Analysis: Explicitly state whether thematic analysis will be conducted solely by the primary researcher or in collaboration with the analysis team. Explain the role of the analysis team in reviewing, refining, and finalizing the coding framework.

3. Discussion:

- Role of Payers in Intervention Development: Acknowledge that payer perspectives were not explicitly included but discuss how reimbursement models and healthcare system factors could influence future implementation.

These have been addressed. The background section now includes details on antibiotic prescribing rates in Ireland and expands on existing POCTs commonly used in Irish primary care. The economic considerations section has been refined to summarize key findings from Fawsitt et al., emphasizing the cost-effectiveness and budget impact of CRP POCT implementation.

 In the methods section, we have clarified that grey literature will be reviewed alongside peer-reviewed sources in the scoping review. Recruitment criteria have been expanded to consider additional demographic factors, such as age and urban versus rural practice settings, to ensure a diverse participant sample. Further clarification has been added regarding qualitative data analysis, specifying the roles of the primary researcher and analysis team in coding and validation.

 The discussion has been revised to address the role of payers in intervention implementation, noting potential reimbursement challenges. These updates enhance the clarity and completeness of the protocol, ensuring alignment with the study's aims and the practical considerations of POCT implementation in Irish primary care.

 Kind regards, 

 Dr Joseph O'Shea
